# Atypical Blue Rubber Bleb Nevus in an African-American Male

**DOI:** 10.1155/2021/9992111

**Published:** 2021-09-17

**Authors:** Harpreet S. Gill, Paul Beinhoff, Sarah Grond, Mohan S. Dhariwal, Pinky Jha

**Affiliations:** Department of Internal Medicine, Medical College of Wisconsin, Wauwatosa, WI 53226, USA

## Abstract

Blue Rubber Bleb Nevus Syndrome (BRBNS), also known as Bean Syndrome, is a rare condition characterized by vascular ectasias that typically present systemically. Most diagnoses are made in early childhood due to cutaneous lesions in Caucasians with familial inheritance. Treatment is usually patient centered due to the wide variance in clinical presentation of the disease. Here, we present a case of BRBNS in a 65-year-old African-American patient with episodic gastrointestinal (GI) bleeding with no previous history. This case emphasizes the need for a higher clinical suspicion of the disease in patients with recurrent GI bleeding.

## 1. Introduction

Blue Rubber Bleb Nevus Syndrome (BRBNS), also known as Bean Syndrome, is primarily a congenital condition that causes multifocal vascular ectasias with frequent systemic manifestations. BRBNS typically presents in early childhood as blue, rubbery vascular lesions of the skin and gastrointestinal (GI) tract, from which the condition gets its name [1]. GI lesions can rupture, leading to subsequent hematochezia and secondary iron deficiency anemia (IDA). This syndrome is exceedingly rare with around 300 cases in the literature to date [2, 3]. Despite the recent increased awareness, treatment and management remains unstandardized due to the diverse presentations of this condition. Our patient had an atypical case of sporadic BRBNS resulting in recurrent jejunal hemorrhages.

## 2. Case Presentation

A 65-year-old African-American man with a past medical history significant for end-stage renal disease since 2018, diabetes type II, hypertension, arteriovenous malformations (AVMs), coronary artery disease, deep vein thrombosis, pulmonary embolism in 2007, heart transplant in 2018 status after myocardial infarction in 2017, hematochezia, and melena for approximately 1 year presented with chronic anemia with episodic acuity for one month. He was on tacrolimus therapy after heart transplant, warfarin for his history of coagulopathy, and monthly, long-acting octreotide injections for his recurrent GI bleeds.

One month prior to his diagnosis of BRBNS, he presented to the ED with recurrent shortness of breath, hematochezia, melena, delirium, and a hemoglobin of 4 g/dL (13.0–17.0 g/dL) and required blood transfusions. He had been noncompliant with his hemodialysis. Other pertinent laboratory findings were platelets: 76 K/uL (165–366 K/uL), creatinine: 15.53 mg/dL (0.70–1.30 mg/dl), and INR: 1.1. The patient was urgently dialyzed. He was normotensive after transfusions, but subsequently became hypotensive and tachycardic.

**Figure 1:**
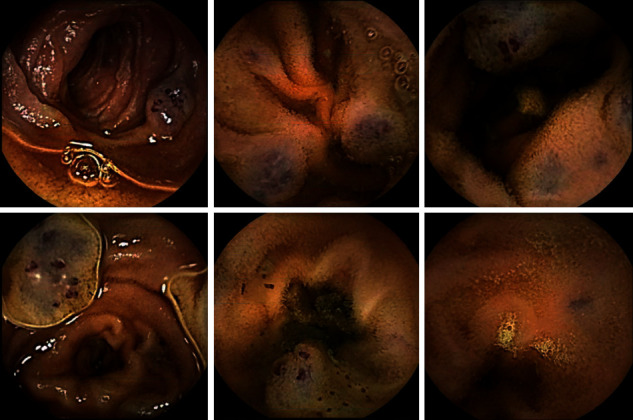
Images from the capsule endoscopy as it progressed through the GI tract display characteristic blue rubber blebs. These vascular ectasias are suggestive of cavernous hemangiomas and AVM. The lesions appear most frequently in the duodenum and gradually decrease in size and density in the distal small bowel.

Gastroenterological studies were completed. The video enteroscopy showed numerous vascular ectasias, thought to be consistent with cavernous hemangiomas. Capsule endoscopy was then conducted, confirming vascular ectasias with superficial dilated venules/capillaries, located throughout the small bowel, as seen in Figure 1. Given these imaging findings and clinical features, BRBNS became the leading differential diagnosis.

**Figure 2:**
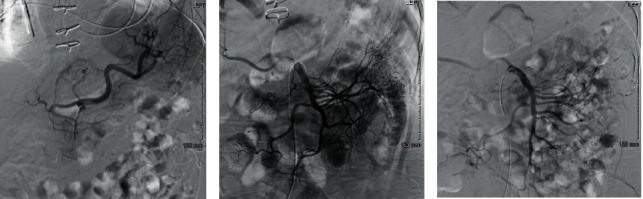
Interventional radiology completed angiography of the celiac (a), superior mesenteric (b), and inferior mesenteric (c) during arterial embolization. 300 to 500 micron PVA particles were used to embolize an AVM in the midjejunum.

Following the study, he was started on everolimus, another immunosuppressant and angiogenesis inhibitor, due to high suspicion of BRBNS. A full-body exam was negative for cutaneous lesions. No biopsies or surgical resections were possible due to the patient's significant bleeding risk. Treatment options were limited given the patient's tenuous hemodynamics and preexisting health conditions. Ultimately, embolization of multiple arterial structures, shown in Figure 2, was performed but was unsuccessful in controlling the bleeding.

Within two months of diagnosis and treatment, the patient and family sought hospice care due to his persistent bleeding.

## 3. Discussion

Blue rubber bleb nevus syndrome (BRBNS) is a rare systemic disease with an incidence around 1 in 14,000 births [4]. The disease typically affects Caucasians more than African-Americans and Asians, with an equal incidence in males and females [5]. Usually, patients are diagnosed at birth or adolescence; however, onset in the 5th and 7th decade of life has been documented [3, 6]. Genetic associations with chromosome 9p abnormalities or angiopoietin endothelial cell tyrosine kinase receptor TIE2 have been described; however, BRBNS can present sporadically [5, 7]. In the case mentioned above, there was no family history related to BRBNS suggesting a sporadic etiology.

GI lesions affect the mucosa at many locations, but usually involve the jejunum and ileum with bleeding and subsequent chronic IDA [3]. Rare, but more complex, GI manifestations include rupture, intussusception, and infection [3]. Disseminated intravascular coagulopathy has also been noted to occur in BRBNS [8, 9]. While concomitant cutaneous and GI manifestations are typical, they can appear separately. As in the presented case, common presentations include hematochezia, melena, or abdominal pain.

Diagnosis is made with endoscopy and imaging studies; however, capsule imaging is the most diagnostically valuable method [10–12]. Of note, BRBNS is diagnosed based on clinical features without requiring positive biopsy findings [5]. The rarity and lack of pathognomonic features of the disease make diagnosis difficult, with the differential for vascular abnormalities including Klippel–Trénaunay–Weber syndrome, Ehlers–Danlos syndrome, Osler–Weber–Rendu syndrome, angiomas, benign tumors, angiosarcoma, Kaposi's sarcoma, angiodysplasias, and Maffucci syndrome [13].

Risk factors for BRBNS are not known beyond limited genetic associations; however, a strong correlation has been noted between upper GI bleeding and hemodialysis (HD). Kuo et al. noted an increased incidence of small bowel bleeding in patients on HD [14]. The purposed pathophysiology of this bleeding is uremia-induced platelet dysfunction [15]. Interestingly, Karagiannis et al. found that small bowel angiodysplasia was identified in 47% of chronic renal failure patients versus 17.6% in control in a study of 68 patients with obscure bleeding, most predominantly in the small bowel [16].

The mortality and management of BRBN is dependent on the number and severity of lesions present [10]. Beyond iron supplementation and transfusions to manage the secondary IDA, treatment is unstandardized [3]. Since 2012, BRBN has been managed medically with low-dose sirolimus [17]. Wong et al. evaluated the use of sirolimus in 23 patients and showed that therapy was well tolerated and nearly unanimous improvement in both cutaneous and gastrointestinal lesions was observed [18].

An alternative therapy with corticosteroids and interferon-alpha has also been described [8]. Improvements of lesion size decreases were mostly temporary with full regrowth of lesions within months following discontinuation. Failure to respond to steroids has been noted with marked improvement following a switch to sirolimus, further supporting its use as a first-line therapy [19].

GI hemangioma treatment includes sclerotherapy, laser therapy, angiographic embolization, surgical resection, and various coagulation methods depending on the morphological features and severity of the lesions. Surgical resection is applicable to localized hemangiomas; however, recurrence of lesions is common [20]. Minimally invasive therapy, as used in the abovementioned case, is favorable as it reduces the risk for ulceration, stricture, and bleeding.

In this presented case, the diffuse nature of the lesions and hemodynamic concerns prohibited the use of laser therapy or surgical resection. The patient was also not a candidate for treatments such as gut transplant due to poor compliance with hemodialysis and self-management of his chronic conditions. This case highlights the need for increased effort and social support in continued care. This patient's extensive medical history and sociological determinants of health significantly impacted his prognosis. We report this case to emphasize atypical presentations of BRBNS and raise awareness for this probably underdiagnosed condition. An early diagnosis of BRBNS and consistent management of preexisting health conditions may have qualified our patient for more aggressive treatment. Further research is warranted to better understand the etiology and pathophysiology of BRBNS that presents in nonpediatric patients without cutaneous involvement.

## Data Availability

All previously used data can be found in the references.
